# Ultra-accurate microbial amplicon sequencing with synthetic long reads

**DOI:** 10.1186/s40168-021-01072-3

**Published:** 2021-06-05

**Authors:** Benjamin J. Callahan, Dmitry Grinevich, Siddhartha Thakur, Michael A. Balamotis, Tuval Ben Yehezkel

**Affiliations:** 1 Department of Population Health and Pathobiology, College of Veterinary Medicine, North Carolina State University, Raleigh, NC USA; 2 Bioinformatics Research Center, North Carolina State University, Raleigh, NC USA; 3 Loop Genomics, San Jose, CA USA

**Keywords:** Synthetic long reads, Amplicon sequencing, Metagenomics, Long-read sequencing

## Abstract

**Background:**

Out of the many pathogenic bacterial species that are known, only a fraction are readily identifiable directly from a complex microbial community using standard next generation DNA sequencing. Long-read sequencing offers the potential to identify a wider range of species and to differentiate between strains within a species, but attaining sufficient accuracy in complex metagenomes remains a challenge.

**Methods:**

Here, we describe and analytically validate LoopSeq, a commercially available synthetic long-read (SLR) sequencing technology that generates highly accurate long reads from standard short reads.

**Results:**

LoopSeq reads are sufficiently long and accurate to identify microbial genes and species directly from complex samples. LoopSeq perfectly recovered the full diversity of 16S rRNA genes from known strains in a synthetic microbial community. Full-length LoopSeq reads had a per-base error rate of 0.005%, which exceeds the accuracy reported for other long-read sequencing technologies. 18S-ITS and genomic sequencing of fungal and bacterial isolates confirmed that LoopSeq sequencing maintains that accuracy for reads up to 6 kb in length. LoopSeq full-length 16S rRNA reads could accurately classify organisms down to the species level in rinsate from retail meat samples, and could differentiate strains within species identified by the CDC as potential foodborne pathogens.

**Conclusions:**

The order-of-magnitude improvement in length and accuracy over standard Illumina amplicon sequencing achieved with LoopSeq enables accurate species-level and strain identification from complex- to low-biomass microbiome samples. The ability to generate accurate and long microbiome sequencing reads using standard short read sequencers will accelerate the building of quality microbial sequence databases and removes a significant hurdle on the path to precision microbial genomics.

Video abstract.

**Supplementary Information:**

The online version contains supplementary material available at 10.1186/s40168-021-01072-3.

## Introduction

The characterization of bacterial species and strains directly from complex microbial samples using amplicon sequencing — in which PCR-amplified DNA fragments (amplicons) from complex genetic mixtures are sequenced — is still an ongoing challenge in microbiology in part due to short sequencing reads not containing enough information to support highly resolved phylogenetic classification. In recent years, the development of long-read sequencing technologies and concomitant advances in their cost-efficiency and accuracy has brought disruptive change to a variety of important biological applications. Long-read sequencing has largely trivialized the generation of complete and accurate de novo bacterial genomes [26], has expanded and improved the enumeration of transcriptional isoforms [5, 37, 38] and immune repertoires [8], and has vastly improved the detection and description of structural genetic variation [30]. Long-read sequencing has become increasingly attractive for amplicon sequencing as well [12, 21, 28]. Long-read amplicon sequencing approaches based on PacBio and Oxford Nanopore sequencing technologies are being developed and deployed in a wide variety of applications (Caskey 2017 [14, 17, 29];), but a combination of error rates, cost, and more limited availability of long-read sequencing capacity continues to impede their widespread application.

Synthetic long-read (SLR) sequencing technologies are appealing because they can leverage inexpensive, accurate, and widely available short-read sequencing platforms such as those from Illumina to generate accurate long-read sequencing data. However, SLR technologies that were previously commercialized by 10x Genomics [40] and Moleculo [27] were not compatible with amplicon sequencing because they assign the same identifier to multiple DNA molecules in the same well/droplet, which is not amenable to reconstructing the sequence of single long molecules. Other SLR methods exist that utilize unique molecular identifiers (UMIs) instead of well/droplet identifiers to tag each DNA molecule with an identifier that can be read by DNA sequencing to identify each molecule, but their chemistries limit their read lengths [9, 23] and these methods have not been commercialized.

We evaluated a new commercially available long-read microbiome sequencing technology that builds upon earlier academic work in SLR sequencing [20, 36]. Specifically, LoopSeq’s SLR chemistry addresses the largest limitation of first [27] and second [40] generation SLR technologies by enabling contiguous short read coverage of single, long DNA molecules. Long DNA molecules are barcoded with UMIs that are then intramolecularly distributed throughout the molecule. After fragmentation, short reads that share the same UMI are used to reconstruct the sequence of the long molecule. Additionally, first [27] and second [40] generation SLR sequencing assigned the same well/droplet barcode to many different DNA molecules and were therefore limited to sequencing molecules with a high degree of dissimilarity, which is not compatible with microbiome amplicon sequencing (e.g., 16S sequencing). LoopSeq’s technology enables the reconstruction of SLRs from mixtures of highly homologous long molecules because UMIs are specific to each input molecule. Even and deep (~30×) short read coverage along UMI barcoded molecules enables an error-correction-by-consensus mechanism that, in principle, could yield very low error rates in the reconstructed long reads.

In this paper, we report on the exceptional accuracy of LoopSeq SLR sequencing in a defined mixture of known bacterial sequences (a mock community) and develop guidelines for filtering and processing LoopSeq SLR sequencing data. We compare LoopSeq full-length 16S rRNA gene amplicon sequencing results to the current gold standard of PacBio CCS (or “HiFi”) sequencing on a common set of human fecal microbiome samples. After denoising, the overall community compositions measured by LoopSeq and PacBio CCS from the same fecal samples were highly concordant. However, LoopSeq achieved higher levels of long-read amplicon sequencing accuracy, and that higher accuracy was likely maintained in complex communities based on the frequencies with which inferred differences between gene variants occurred in the conserved or variable regions of the 16S rRNA gene. Finally, we show how LoopSeq full-length 16S sequencing can be used to identify CDC defined foodborne pathogen species from samples of US retail meat and to distinguish distinct strains within those species.

## Results

### Accuracy and error modes: 16S sequencing of the Zymo mock community

The ZymoBIOMICS Microbial Community DNA Standard (the Zymo mock community) consists of genomic DNA from 8 bacterial strains of the species *Bacillus subtilis*, *Enterococcus faecalis*, *Escherichia coli*, *Lactobacillus fermentum*, *Listeria monocytogenes*, *Pseudomonas aeruginosa*, *Salmonella enterica*, and *Staphylococcus aureus*. We used the LoopSeq 16S Long Read Kit (Loop Genomics, CA) to barcode and amplify the full-length 16S rRNA gene (the “Methods” section), which was then sequenced by Loop Genomics using Illumina NextSeq500 PE150. The assembled long reads were filtered to remove those that did not contain both primers, which removed ~15% of the reads. Then reads with lengths outside the expected range (1400-1600 nts) or that contained more than two expected errors according to their quality scores [13, 18] were filtered out, removing another ~3% of the reads. The ~83% of reads that passed filtering were processed by the DADA2 method using the current 1.18 release version and default parameters (the “Methods” section) to produce a set of denoised amplicon sequence variants (ASVs) discriminated at single-nucleotide resolution [11].

We conclude that all 27 denoised ASVs represent true sequences without any residual errors, using the same evaluation approach previously described for PacBio long-read amplicon sequencing [12]. In short, 26 of the 27 denoised ASVs from the Zymo 16S rRNA data were exact matches to previously sequenced genomes of the expected species. The sole exception was a single *L. fermentum* ASV with one mismatch from the previously sequenced variants available in the NCBI’s nt database. Multiple ASVs were detected from six of the eight mock community strains, including *L. fermentum*. In each case, these variants appear in the integer ratios consistent with being different alleles of the known number of 16S rRNA genes in the genomes of those strains (Figure S1). No contaminant sequences (i.e., sequences originating from outside the mock community) and no false positive sequences (i.e., sequences containing uncorrected errors) were present in the denoised ASVs.

**Fig. 1:**
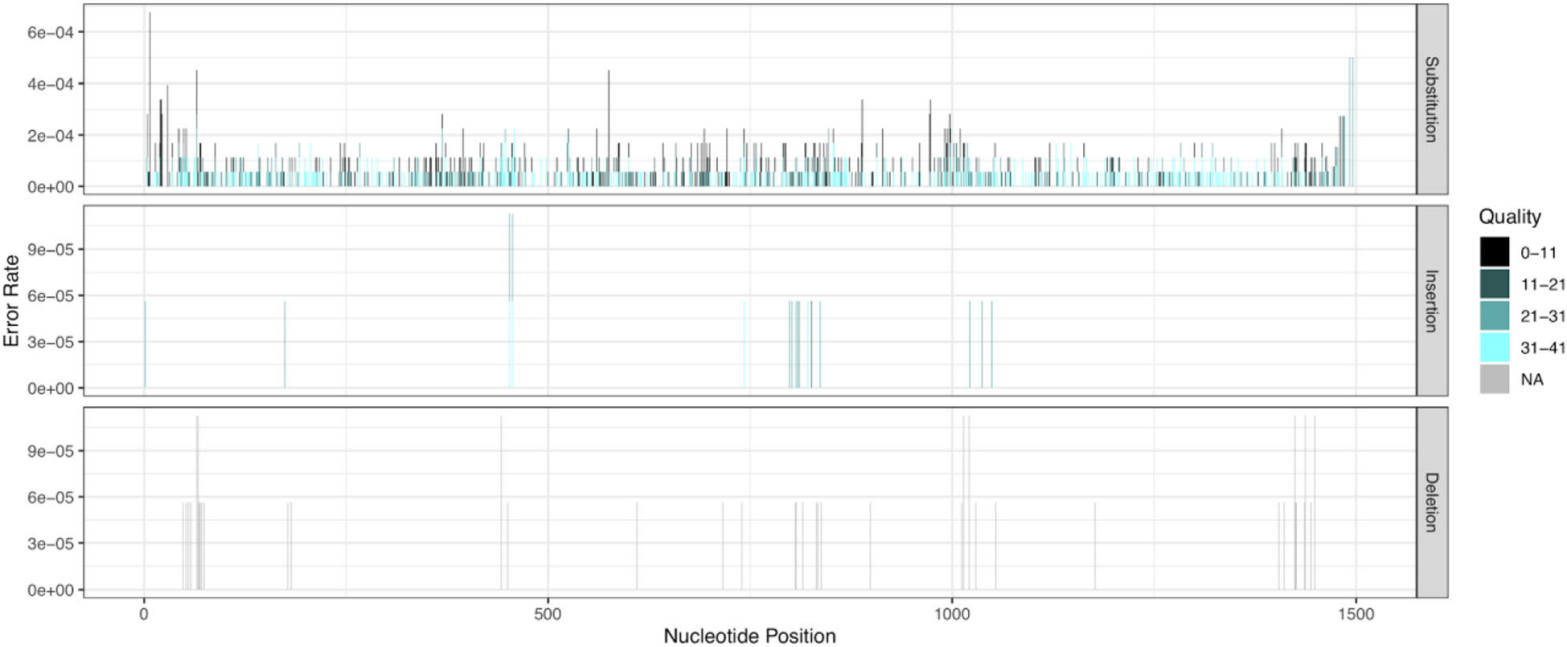
The rates of LoopSeq point errors by position in read, type (substitution/insertion/deletion), and quality score. The scale of the y-axis is different for substitutions because they occur at a much higher rate than insertions or deletions

LoopSeq long amplicon sequencing reads were highly accurate. In total, 94.6% of these ~1500 nt reads contained no errors at all, a larger fraction than the ~50-90% of error-free reads in standard Illumina short reads given that technology’s per-base error rates of 0.1-0.24% and read lengths of 100-300 nucleotides [33]. The DADA2 denoising method [10] was used to associate error-containing LoopSeq reads to the true sequence from which they most likely originated, and the locations, type, and quality score associated with each point error were recorded (the “Methods” section, Fig. 1). Insertion and deletion errors were extremely rare (< 2 × 10^−6^ per nucleotide) and there was no evidence of specific read positions associated with significantly higher insertion or deletion error rates. Substitution errors were somewhat more common (4.6 × 10^−5^ per nucleotide) and occurred at a slightly higher rate near the start and end of the reads. This was predicted by lower quality scores and expected from the lower coverage of the long-read contigs by the short-reads at the ends of the contigs. Overall, this per-nucleotide error rate of ~5 × 10^−5^ per nucleotide, alternatively expressed as a 99.995% per-nucleotide accuracy, significantly exceeds the best per-base accuracy results currently reported in the literature for amplicon sequencing using standard Illumina sequencing or Pacific Biosciences CCS long-read sequencing [12, 33].

**Fig. 2:**
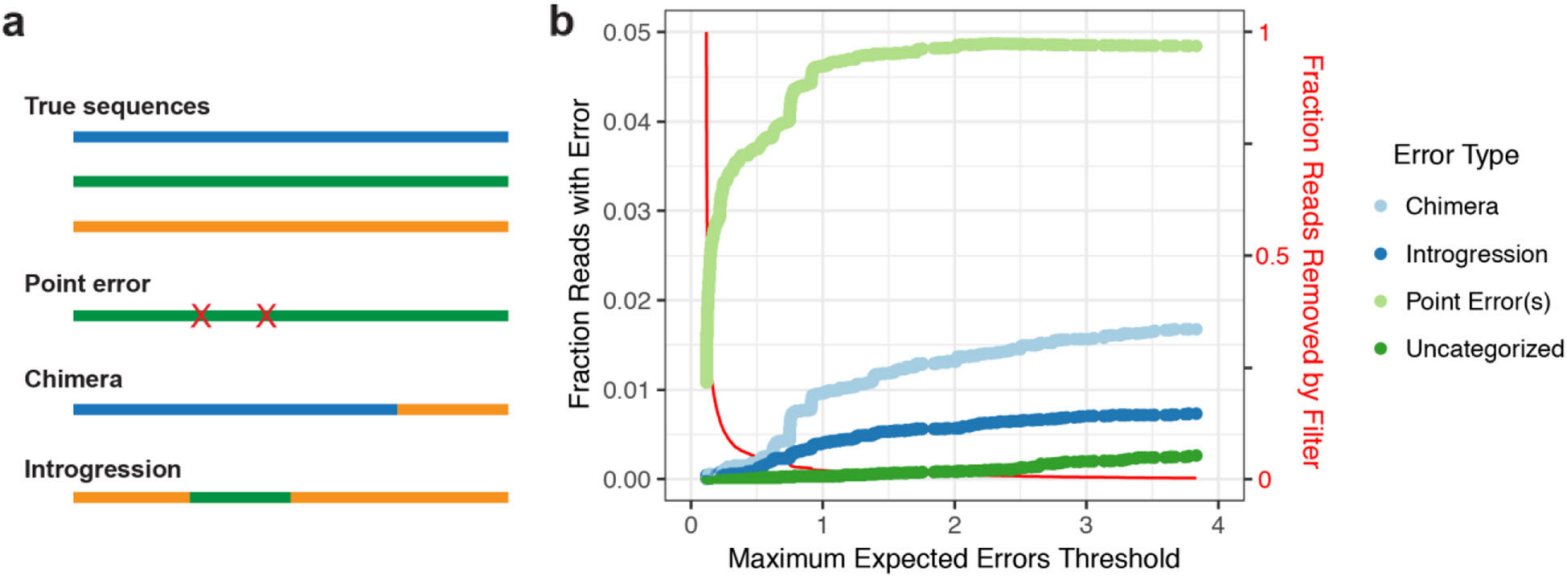
Error types and rates in LoopSeq data. (**a**) Schematic description of three error types: point errors, chimeras, introgressions. (**b**) The fraction of each error type as a function of expected error quality filter threshold. The red line shows the fraction of LoopSeq reads removed as a function of the filter threshold

We performed a further manual inspection of potential structural errors in LoopSeq amplicon sequencing data, for example, PCR chimeras that are formed through processes that modify large segments of the sequencing read rather than a single position at a time. We identified a type of structural error we refer to as an introgression, in which a segment of one amplicon is replaced (or is introgressed into) the homologous segment in another amplicon (Fig. 2a). This error mode arises due to an interaction between PCR chimeras and the assembly of short-read into an SLR. The presence of early-round PCR chimeras can result in a segment of chimeric DNA being selected by the assembler when constructing the long-read sequence from all of the short-reads sharing that UMI. Usefully, lower quality scores are typically found in the introgressed segment, reflecting the lower level of short-read consensus at those positions.

We developed a sliding window approach to identify structural errors, both typical PCR chimeras and introgressions, in the uncorrected LoopSeq reads from this mock community of known composition (the “Methods” section). For each read, we determined whether it was correct (i.e., contained no errors) or incorrect. If it was incorrect, we determined whether it was a chimera, an introgression, or contained point errors. The fraction of reads of each of these error types is plotted as a function of the expected errors filtering threshold (Edgar 2017) [18] for the unfiltered LoopSeq reads from the Zymo mock community in Fig. 2b. A small number of incorrect long reads could not be unambiguously categorized, perhaps because they were structural errors that also contained point errors.

Structural errors (chimeras and introgressions) accounted for just over 2% of the reads in the unfiltered LoopSeq amplicon reads from the Zymo mock community, but this fraction significantly decreased with stricter quality filtering (Fig. 2b). For this sequencing library, a threshold of 0.5 maximum expected errors appeared to effectively balance the removal of reads by the filter with the suppression of structural errors. We explored the effects that this optimized filtering had on subsequent denoising by DADA2 (the “Methods” section). We found that optimized filtering allowed DADA2’s singleton detection (DETECT_SINGLETONS=TRUE) and a more sensitive ASV detection threshold (OMEGA_A=1e−10) to be used to denoise LoopSeq data: Only 4 false positive denoised ASVs were identified, each represented by a single read and that in total accounted for < 0.03% of all denoised reads. New methods for de novo identification of introgressions could largely eliminate those remaining rare false positives. This suggests the possibility of long-read amplicon sequencing that simultaneously achieves maximum (single-nucleotide) resolution, maximum (singleton) sensitivity to rare variants, and a near-zero false positive rate.

### Accuracy of longer reads: 18S-ITS and genomic sequencing of fungal and bacterial isolates

**Fig. 3:**
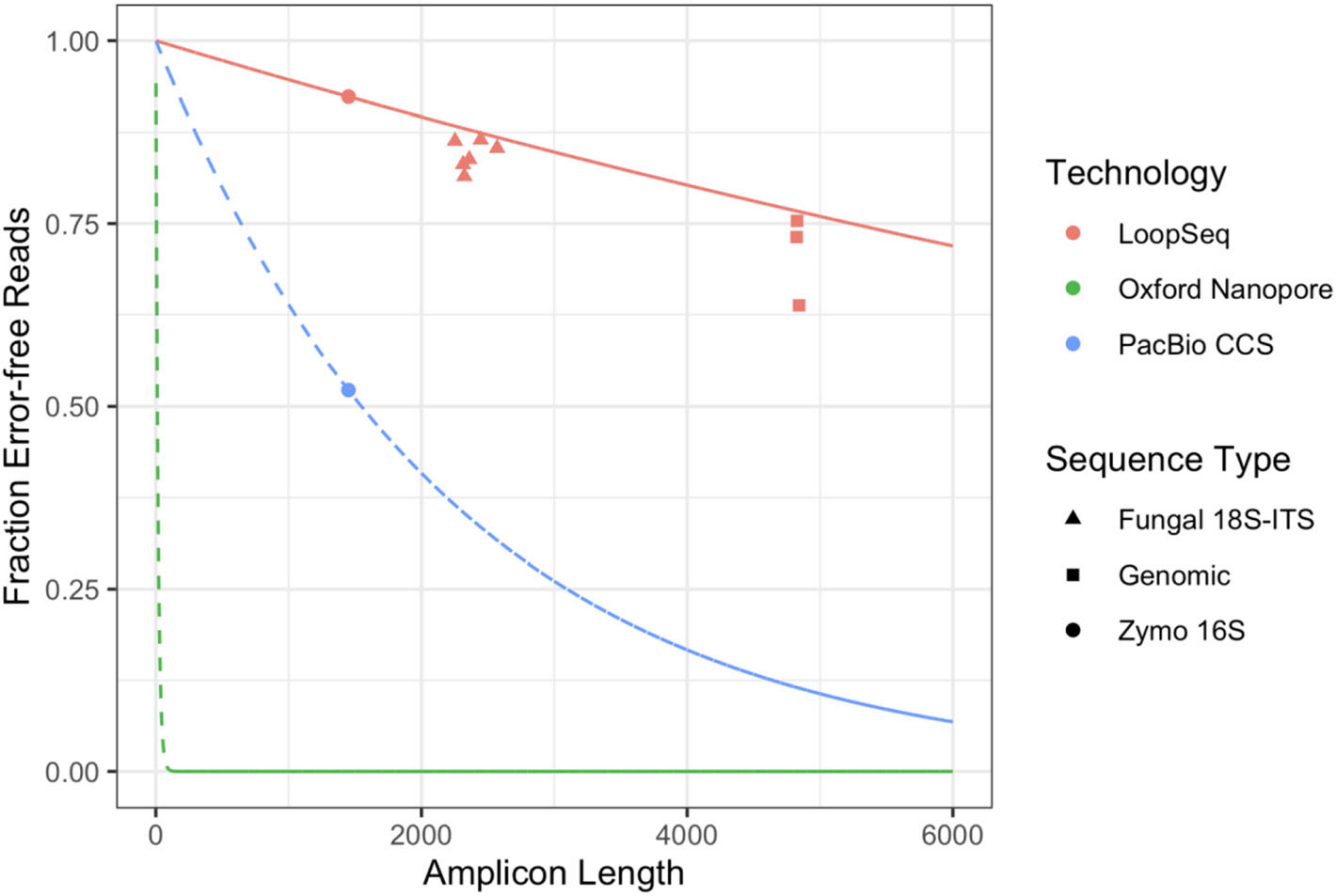
The fraction of error-free amplicon sequencing reads of different lengths using commercially available long-read sequencing technologies. Points represent observations from measurements of defined communities (the Zymo mock community) or single-strain isolates (fungal and bacterial isolates) that were reported in this manuscript for LoopSeq, and that were reported in [12] for PacBio CCS reads. The 16S rRNA and fungal 18S-ITS data are from traditional amplicon sequencing of a single genetic region (the “Methods” section). The LoopSeq genomic amplicon data is based on LoopSeq sequencing of randomly amplified regions of the genome of several bacteria (the “Methods” section). Lines represent the expected error-free fraction based on measurements of the error rates in 16S rRNA gene amplicon sequencing data, and assuming a constant per-base error rate. The Oxford Nanopore line is based on the per-base error-rate of 6% reported by the manufacturer for the R10 chemistry

We performed LoopSeq amplicon sequencing of the approximately 2.3 kb 18S-ITS gene region from isolates of six fungal species obtained from the ATCC: *Saccharomyces cerevisiae*, *Aspergillus oryzae*, *Candida albicans*, *Trichoderma reesei*, *Kluyveromyces lactis*, *Penicillium chrysogenum*. LoopSeq reads were filtered for the presence of the forward and reverse primers and then trimmed to the region between the primers. In each sample, over 80% of the reads had identical sequences that also exactly matched the 18S-ITS gene region from a previously sequenced isolate of that fungal species. We determined these reads to be the error-free fraction of the data (Fig. 3). This may be a slight underestimate if some reads with different sequences represented error-free reads from minority alleles of the many (100s) of copies of the 18S-ITS gene region present in some fungi.

We also performed LoopSeq sequencing of randomly amplified segments of the genomes from isolates of three bacterial species obtained from the ATCC: *Nitrosomonas europaea*, *Desulfovibrio desulfuricans*, and *Salinispora tropica*. LoopSeq reads were filtered for length between 4000 and 6000 bases, resulting in a median read length of ~5 kb. The error-free fraction was determined to be those reads that exactly matched the associated reference genome. The error-free fraction of these LoopSeq reads was over 60% in all three species (Fig. 3). This may be a slight underestimate if errors exist in the reference genomes, or if non-genomic elements such as plasmids were present in the sequencing data.

### Performance in complex communities: human fecal samples

We performed LoopSeq full-length 16S amplicon sequencing of the DNA extracted from three human fecal samples. The same extracted DNA had been previously characterized by PacBio full-length 16S amplicon sequencing [12]. The raw LoopSeq data was filtered and processed by the DADA2 method using default parameters (the “Methods” section). The ASV tables produced by LoopSeq and by PacBio (two replicates, using two versions of their Sequel chemistries) were merged into a common ASV table (the “Methods” section).

**Fig. 4:**
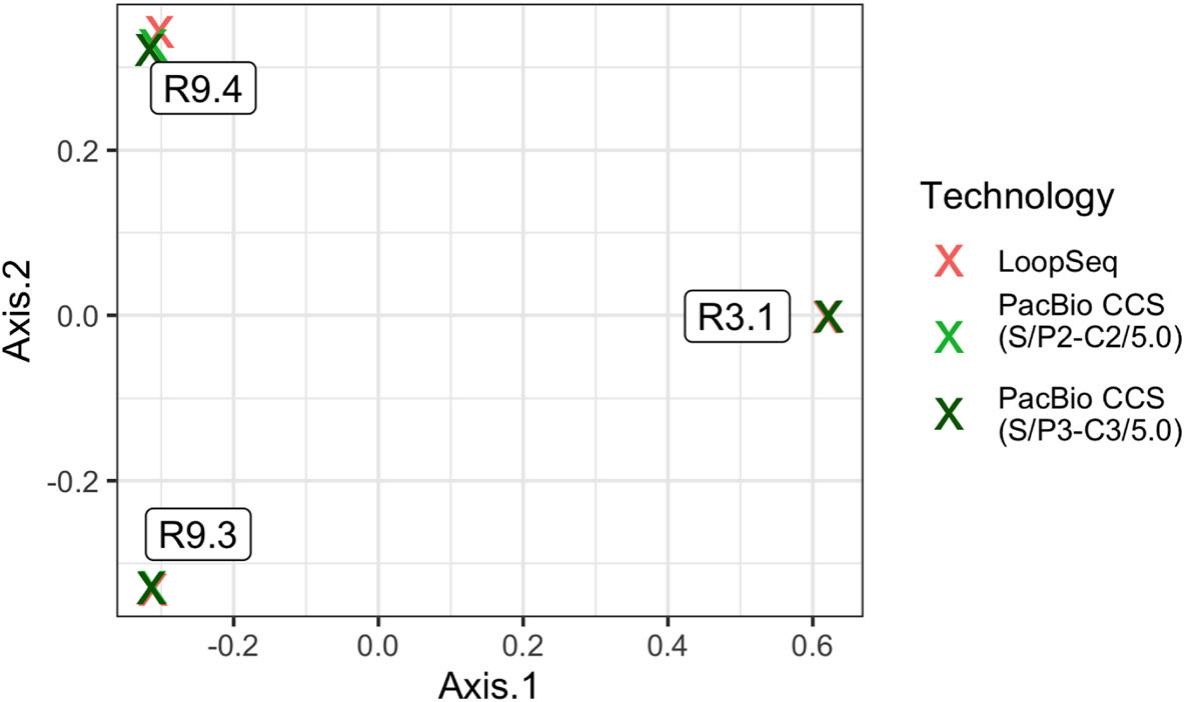
PCoA ordination of the total community compositions of three human fecal samples (R3.1, R9.3, R9.4) as measured by LoopSeq and PacBio full-length 16S rRNA gene sequencing. The community compositions measured by each technology were highly similar, leading to the data points on this ordination being highly overlapping for each sample. PacBio CCS measurements were made using two different sequencing chemistries as indicated in the legend parentheticals

The communities measured by LoopSeq and PacBio were highly concordant. A median of 89.9% of the reads detected by LoopSeq in each sample shared the same sequence with reads also detected by PacBio, while the same measure between the PacBio replicates was 94.1%. We used the Bray-Curtis metric to quantify community-wide dissimilarity between the measured communities in each sample by each technology. Visualization of those results in a PCoA ordination plot (Fig. 4) revealed that differences between LoopSeq and PacBio measurements of the same sample were trivial compared to differences between samples. In fact, the median Bray-Curtis dissimilarity between LoopSeq and PacBio measurements of the same sample was just 0.187, barely higher than the median 0.173 Bray-Curtis dissimilarity between replicate PacBio measurements of the same sample.

**Fig. 5:**
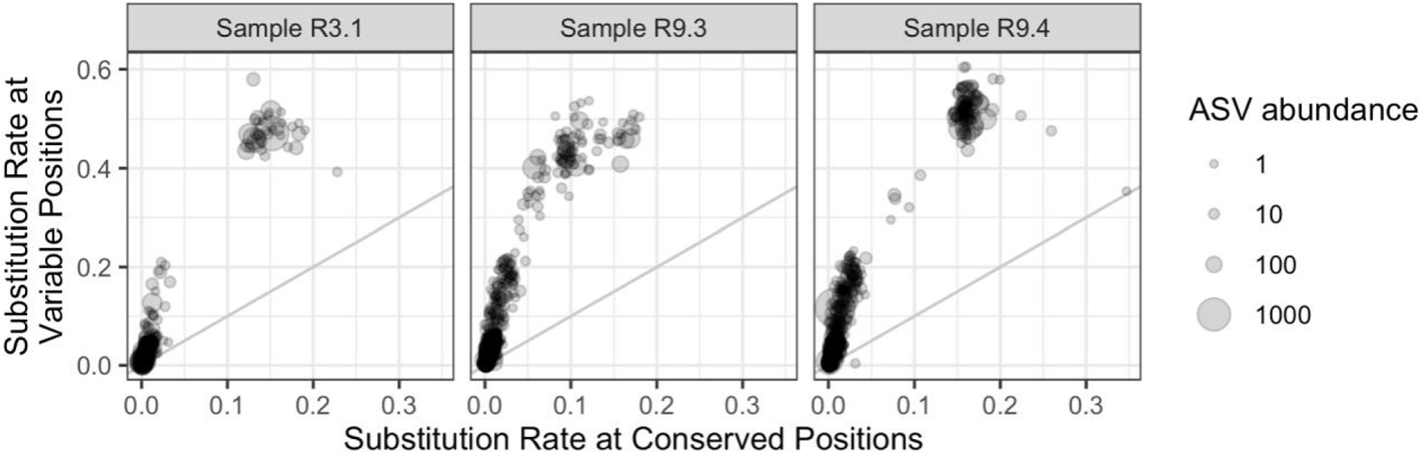
The rate of substitution differences between DADA2-denoised ASVs and the next closest ASV that are in conserved vs. variable regions of the 16S gene, in LoopSeq data from three human fecal samples. If substitution patterns were random (as would be expected if they were caused by sequencing errors) then points should fall along the gray line of equal rates

The differences between LoopSeq ASVs were highly enriched at known variable positions of the 16S rRNA gene, supporting high LoopSeq accuracy in the human fecal samples. We performed high-sensitivity sample inference on these human fecal samples using the DADA2 method (the “Methods” section) which also provides a full description of the substitutions between each ASV and the “sibling” ASV from which it was distinguished by the denoising algorithm. We used the ssu-align program (the “Methods” section) to define whether substitutions between sibling ASVs occurred at conserved or variable regions of the 16S rRNA gene [31]. The results of this analysis for each ASV identified by DADA2 in high-sensitivity mode are shown in Fig. 5.

Substitution differences between sibling LoopSeq ASVs occur at approximately a fourfold higher rate in the variable positions of the 16S rRNA gene than they do in conserved regions. If sequence diversity was driven by sequencing errors, these substitutions should occur at an equal rate in conserved and variable regions. This pattern supports the high accuracy of LoopSeq long-read amplicon sequencing in complex community samples, consistent with the results on the simpler mock community.

### Example application: identifying foodborne pathogen species in retail meat

To investigate the potential for ultra-accurate long-read 16S sequencing to identify and track foodborne pathogens, we performed LoopSeq 16S sequencing on DNA extracted from a rinsate of six samples of US retail meat (the “Methods” section). We were particularly interested in the foodborne pathogen species that the CDC has identified as of particular importance in retail meat: *Yersinia enterocolitica*, *Escherichia coli*, *Salmonella enterica*, *Clostridium perfringens*, *Campylobacter* spp*.*, and *Listeria monocytogenes*. We performed high-sensitivity sample inference on these retail meat samples using DADA2, and assigned taxonomy down to the genus level using the naive Bayesian classifier method and the Silva database (the “Methods” section). Denoised ASVs with genus assignments that matched high-interest foodborne pathogen species were then given species assignments if their BLAST results unambiguously supported a particular species (the “Methods” section).

**Fig. 6:**
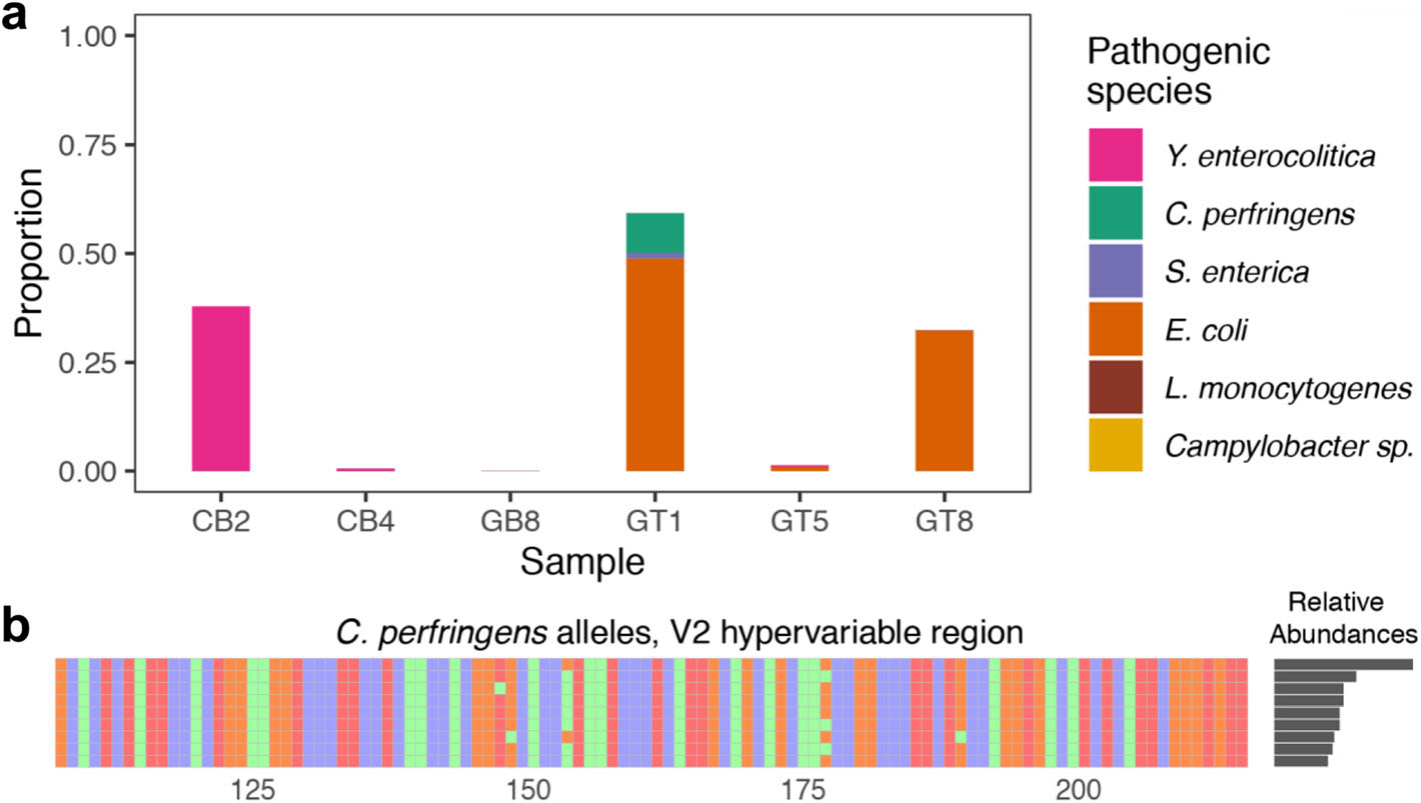
Foodborne pathogen species detected in retail meat rinse samples. (**a**) The species and relative abundance of six common foodborne pathogen species were determined from full-length LoopSeq 16S sequencing of six retail meat samples. *Campylobacter* and *Listeria monocytogenes* did not appear in these samples. (**b**) Nine distinct alleles of the 16S rRNA gene were detected in the *C. perfringens* strain present in sample GT1. The first allele was present at roughly twice the abundance of the others, consistent with it having two copies in the genome while the rest have only one copy. *C. perfringens* has 10 copies of the 16S rRNA gene in its genome. Only the V2 region is shown for visual simplicity

The accuracy and length of the full-length LoopSeq 16S sequences allowed us to distinguish the foodborne pathogen species of interest (Fig. 6a, the “Methods” section). The full-length 16S sequences from *Yersiania* were clearly distinguishable as belonging to *Y. enterocolitica*, and no other closely related *Yersinia* that are not typically foodborne pathogens, including the notorious metagenomic false positive *Y. pestis* [1–3]. *C. perfringens* sequences were distinguishable from the closely related *C. septicum*, and the *Salmonella* sequences in sample GT5 were all unambiguously identified as *S. enterica* subsp. *enterica.*

Most bacteria have multiple ribosomal operons, and the complete set of 16S rRNA gene allelic variation of many of the abundant strains of the foodborne pathogen species present in these retail meat samples was fully resolved (e.g., Fig. 6b). However, in samples where multiple related strains of the same species were present, as was the case for *E. coli* in samples GT1 and GT8, it was not always possible to unambiguously separate ASVs into strain-level bins, and the full allelic complement could not be captured for low abundance strains. Using a previously described strategy [12], we used the full complement of 16S alleles to determine that the *S. enterica* strain in sample GT1 most closely matched a previously sequenced genome from the pathogenic Newport serovar, warranting further investigation into its potential pathogenicity. This direct inference could be made possible in the future with a better understanding of the phylogenetic coherence of the Newport serovar, and a more complete catalog of high-quality *Salmonella* genomes from pathogenic and non-pathogenic strains.

## Discussion

Amplicon sequencing is a cornerstone method in the life sciences, notably used for the characterization of microbial diversity in complex samples. The combination of PCR amplification and subsequent sequencing massively enriches a targeted genetic locus and provides detailed information about the genetic diversity at that locus. A fundamental constraint on amplicon sequencing has been the short read lengths of modern high-throughput sequencers, but that constraint has been overcome by the rise of long-read sequencing technologies. In a world of expanding sequencing options, it is critical to understand the accuracy and economics of long-read amplicon sequencing technologies in order to know when specific technologies are the right choice for a given application.

Commercially available synthetic long-read (SLR) sequencing technologies — in which long sequencing reads are reconstructed from short reads containing molecular tags indicating origination from a common DNA molecule — have been around for a number of years, but not for amplicon sequencing where homology between sequences is high. The LoopSeq technology recently commercialized by Loop Genomics applies unique molecular identifiers (UMIs) to each DNA molecule, and allows precise and accurate long-reads to be constructed from amplicon sequencing data. Here, we showed that LoopSeq amplicon sequencing attains higher accuracy than previously reported for commonly used amplicon sequencing technologies, can scale out to sequence lengths of at least 6 kb while maintaining very high accuracy, and can be used to precisely survey the composition of complex microbial communities.

Accuracy is essential to many amplicon sequencing applications, and the level of accuracy achieved by LoopSeq may open up new opportunities. Long-read amplicon sequencing using the PacBio and Oxford Nanopore technologies ([12]; Eren 2019 [24];) has received increased recent attention, with encouraging results demonstrating that per-base accuracy exceeding common short-read approaches can be obtained by combining long-read sequencing with molecular methods such as the construction of PacBio circular consensus (CCS) reads and appropriate bioinformatics. In the bacterial profiling application, multiple studies have shown that substantial improvements in species and subspecies resolution can be achieved by sequencing the entire ~1.5 kb 16S gene, rather than just segments of 100-500 bases as is most commonly practiced today, and that even greater resolution is achievable by extending the sequenced region to most or all of the *rrn* operon [30]. The LoopSeq per-base accuracy of 99.995% we observed here suggests that this technology should also be considered for high-resolution long-read amplicon sequencing applications.

The new frontier of amplicon sequencing beyond 1.5 kb may be where the accuracy attainable by LoopSeq is most important. Using the manufacturer-recommended coverage thresholds and a common bioinformatics workflow, we showed that > 90% of LoopSeq full-length 16S reads were error free as compared to ~ 50% error-free full-length 16S PacBio CCS reads [12]. Fifty percent is a sufficiently high fraction of error-free reads for modern denoising methods to achieve single-nucleotide resolution with high accuracy, and thus the total community compositions determined after denoising full-length 16S rRNA gene sequences obtained using LoopSeq reads and PacBio CCS reads from the same human fecal samples were very similar (Fig. 4). However, if we consider amplicon sequencing of the entire ~5 kb *rrn* operon, then LoopSeq produces > 50% error-free reads, while only ~10% of PacBio CCS reads would be expected to be error-free, limiting the resolution of lower-abundance members of a sampled community. Single-nucleotide resolution with high accuracy from the entire *rrn* operon opens the door for population-level analysis of genetic diversity, rather than just comparisons among species, and would enhance discrimination of critical sub-species variation such as pathogen and non-pathogen clades, especially in sample types where alternative shotgun metagenomics methods are challenged by large amounts of non-target DNA.

PCR amplicon chimeras, in which sequencing reads are produced, that are a combination of multiple true DNA molecules, can be particularly pernicious for the accurate reconstruction of complex communities. LoopSeq SLRs have a significant advantage over standard long-read amplicon sequencing approaches in this regard because LoopSeq SLRs are assembled from a consensus of UMI-tagged reads. As a result, chimeric molecules do not contribute to the consensus assembly unless the chimera formed at the first cycle or two of PCR causing chimeric reads to constitute a majority of the short reads for that UMI. Chimera rates are also at their lowest during early PCR cycles, further reducing the effective LoopSeq chimera rate. In standard amplicon sequencing technologies, chimeric molecules formed during all PCR cycles will be present in the final data. This potential to leverage UMIs to identify and remove SLR chimeras was also demonstrated previously in a different SLR technology [9].

We identified and described an SLR-specific structural sequencing error, the introgression, in which a long SLR is formed with an internal insertion of a short segment from another DNA molecule (Fig. 2a). Introgressions are caused by the stochastic preponderance of reads from chimeric DNA molecules over short regions of the SLR. The rate of introgressions in LoopSeq data is low, and no introgressions were detected when typical denoising of the Zymo mock community was performed that screened out singletons. Standard quality filtering based on expected errors further reduced the fraction of introgressions in the raw data (Fig. 2). As LoopSeq or other SLR sequencing technologies become more widely used it may be useful to revisit the quality filtering approaches used for such data. Dips in the quality scores corresponding to introgressed LoopSeq regions suggest that sliding window quality screens may be a useful tool for such data.

In this manuscript, we focused on comparing LoopSeq to the long-read sequencing technologies developed by PacBio and Oxford Nanopore (ONT), because those technologies are currently the most widely used and are already commercially available. However, an important current research direction is the marriage of UMI methods with those long-read sequencing technologies to improve their accuracy. Recently, Karst and colleagues described and evaluated such a method, and reported achieving accuracy comparable to that reported here by pairing UMIs with Oxford Nanopore sequencing, and even higher levels of accuracy from pairing UMIs and PacBio CCS sequencing [24]. We expect this general approach, if not the exact implementation in Karst et al., will prove to be an attractive option for highly accurate long-read amplicon sequencing as it continues to develop. Each of these high-fidelity long-read sequencing methods (LoopSeq SLRs, PacBio + UMIs, ONT + UMIs) are able to achieve very high accuracy (> 99.99% per-base) that can be increased further at the expense of throughput by increasing the number of reads per UMI, with the exception that ONT may experience an accuracy plateau due to continuing issues with systematic error modes [24]. It is likely that in the next few years different highly accurate long-read technologies will find unique niches within microbial genomics. For example, ONT sequencing can be advantageous for field applications of microbial genomics in which rapid diagnosis is key while LoopSeq and PacBio HiFi long reads might be more useful for applications that require high accuracy and that can be obtained within days, not hours.

A balance between the cost efficiency of high-fidelity long-read sequencing methods and the value of sequencing accuracy in different applications will determine their ultimate impact. The LoopSeq data presented in this manuscript used an average of 30× Illumina short-read coverage of each SLR, which naively extrapolates to a 30× increase in per-base sequencing cost relative to short-read amplicon sequencing. However, LoopSeq works with 150 nt paired-end reads without any cost to SLR length or quality, and 150 nt reads are up to tenfold cheaper per-base than the longer 300 nt paired-end reads currently in common usage for short-read amplicon sequencing. A cost comparison with other high-fidelity long-read sequencing methods is complicated by the rapid technological progress in this area, and the future cost evolution of the short-read (LoopSeq), PacBio, and ONT sequencing technologies underlying different methods is unclear. Shallow shotgun sequencing is another alternative suitable for profiling complex microbial communities that are often performed with similar short-read library sizes (~1-2 million) as might be generated for a LoopSeq 16S rRNA gene library. If large amounts of non-target DNA are present in relevant samples, then the targeting of sequencing effort by amplicon sequencing will make LoopSeq more cost effective for community profiling, but shotgun sequencing allows additional information on functional potential to be gleaned.

The rapidly increasing accuracy and length of available amplicon sequencing technologies has laid bare the limitations of commonly used taxonomic assignment methods for 16S rRNA gene data. There are fundamental limits on the taxonomic resolution available from any marker-gene sequencing approach, but the most widely used methods for taxonomic assignment from 16S sequences were developed for short-read data and often do not even attempt to make taxonomic assignments beyond the genus level. Long-read amplicon sequencing data of the accuracy achieved by LoopSeq allows for species-level assignment from 16S rRNA gene data in most cases. Even higher levels of sub-species resolution are achievable, but substantial roadblocks exist in practice due to the multi-copy nature of the *rrn* operon in bacteria [25]. Full-length 16S rRNA gene sequencing with single-nucleotide resolution will resolve all intragenomic variation between the 16S alleles carried by a single bacterium [12, 21], but there is no currently automated way to reconstruct the bins containing alleles arising from a common genome. There is also no universal database that contains and labels the full complement of 16S rRNA gene alleles arising from each strain. Furthermore, many reference genomes created from short-read sequencing data do not resolve the multiple copies of the *rrn* operon at all. Preliminary evidence suggests that, at least in some cases, pathogenic and non-pathogenic *E. coli* can be distinguished from full-length 16S sequencing alone [12], but current taxonomic assignment methods do not approach that level of resolution.

There is a much larger potential universe of applications for LoopSeq and other highly accurate long-read sequencing technologies beyond profiling microbial communities by sequencing the *rrn* operon. One topical example is viral genomic sequencing, such as that being applied to the population genetics and genomic epidemiology of the SARS-CoV-2 virus. Viral DNA exists as a tiny minority of the DNA in clinical samples, and common SARS-CoV-2 sequencing approaches rely on amplifying nearly 100 different genetic regions that are then stitched together to reconstruct a consensus genome sequence [16]. Highly accurate long-read amplicon sequencing opens the door to simpler protocols using far fewer primers, and that can also achieve long-range linkage information [6, 19, 35]. These same advantages in simplicity, accuracy, and long-range linkage information are driving the adoption of highly accurate long-read amplicon sequencing for the study of HIV [14, 32], HLA/MHC [22, 34, 39], and oncogene diversity in solid tumors [15].

## Conclusion

Three aspects of microbial genomics will have significant bearing on bringing about a future of precision microbiology: the (1) accuracy of reading microbial genomes, the (2) discriminatory power of microbial sequencing reads, largely determined by read lengths, and the (3) quality of microbial sequence databases. Improvements in accuracy and length will feed directly into building better databases and generate a positive feedback loop that will eventually trivialize microbial identification and characterization. In this manuscript, we showed how short-read sequencers can be used to generate microbial DNA reads with a combination of length and accuracy that matches and surpasses currently available methods. This LoopSeq technology leverages already widely available short read sequencers and is commercially supported, a combination of attributes that could accelerate the uptake of accurate long-read sequencing in general. Amplicon sequencing that is an order-of-magnitude longer and an order-of-magnitude more accurate than the Illumina short-read standard is available today. We look forward to the ways this technology will be applied in the future.

## Methods

### Samples and DNA extraction

#### Zymo mock community

The ZymoBIOMICS™ Microbial Community DNA Standard (P/N: D6306, Lot ZRC190811) was obtained from the manufacturer Zymo Research (Irvine, CA). The Zymo mock community contains genomic DNA from eight phylogenetically diverse bacteria, and two yeast strains not amplified by our 16S rRNA gene amplicon sequencing protocol. Note that five strains in ZymoBIOMICS™ standards were replaced with similar strains in Lot ZRC190633. The sample analyzed here is from a post-replacement lot.

#### Fungal isolates

Extracted and purified genomic DNA from the following six fungal isolates were obtained from the ATCC: *Saccharomyces cerevisiae Meyen ex E.C. Hansen* (Catalog Number ATCC 201389D-5), *Aspergillus oryzae var. oryzae* (Catalog Number ATCC 42149D-2), *Candida albicans* (*Robin*) *Berkhout* (Catalog Number ATCC10231D-5), *Trichoderma reesei Simmons* (Catalog Number ATCC 13631D-2), *Kluyveromyces lactis* (*Dombrowski*) *van der Walt* (Catalog Number ATCC 8585D-5), and *Penicillium chrysogenum Thom* (Catalog Number ATCC 10106D-2).

#### Bacterial isolates

Extracted and purified genomic DNA for the following three bacterial isolates were obtained from the ATCC: *Nitrosomonas europaea* (Catalog Number *ATCC 19718D-5*): https://www.ncbi.nlm.nih.gov/nuccore/AL954747, *Desulfovibrio desulfuricans* (Catalog Number *ATCC 27774D-5*): https://www.ncbi.nlm.nih.gov/nuccore/CP001358, and *Salinispora tropica* (Catalog Number ATCC *CNB-440D-5*): https://www.ncbi.nlm.nih.gov/nuccore/CP000667.

#### Human fecal samples

Genomic DNA was obtained from three human fecal samples previously analyzed in a publication on PacBio long-read amplicon sequencing [12]. The aliquots of DNA analyzed here were extracted as part of, and as described in, that publication.

#### Retail meat rinse samples

Two hundred fifty milliliters buffered peptone water (BPW) was added to > 50 g of retail meat (bone-in, skin on chicken breast; ground turkey, ground beef < 85% lean; or bone-in pork chop), and samples were shaken at room temperature for 15 min at 250 rpm. Meat samples were incubated 18 h in BPW at 37 °C. Two milliliters rinsate was centrifuged at 1000×*g* for 5 min, and supernatant was discarded. DNA was isolated from pellets using Lucigen MasterPure™ Gram Positive DNA Purification Kit according to manufacturer protocols. DNA was quantified by Qubit dsDNA HS Assay Kit assessed for purity using a Nanodrop 2000c (ThermoFisher Scientific).

### Sequencing library preparation

Sequencing libraries were prepared from extracted genomic DNA with the commercially available LoopSeq kits from Loop Genomics (protocols available at loopgenomics.com). Synthetic long reads (SLRs) were constructed from the short-read sequencing reads using the standard Loop Genomics informatics pipeline. The process involves attaching two DNA tags: one Unique Molecular Identifier (UMI) to each unique “parent” molecule and one sample-specific tag (i.e., a sample index) equally to all molecules in the same sample. Barcoded molecules are amplified, multiplexed, and each UMI is distributed intramolecularly to a random position within each parent molecule. Molecules are then fragmented into smaller units at the position of each UMI, creating a library of UMI-tagged fragments with an average length of 400 bp compatible with an Illumina sequencing platform run in PE150 mode.

#### Full-length 16S sequencing

For each LoopSeq Microbiome 16S kit, up to 24 samples were processed in multiplex and ~12,000 1.5 kb molecules were sequenced per sample (~300 k molecules from a complete kit run). 100-150M PE150 reads (50-75M clusters passing filter) were used for each sequencing run, yielding ~20 gigabases (Gb) of data. The complete sample preparation and sequencing protocol can be found in this link.

#### Full-length 18S-ITS sequencing

For each LoopSeq Mycobiome 18S-ITS kit, up to 24 samples were processed in multiplex and ~12,500~2.3 kb molecules were sequenced per sample (~300 k molecules from a complete kit run). 175-250M PE150 reads (87.5-125M clusters passing filter) were used for each sequencing run, yielding ~35 gigabases (Gb) of data. The complete sample preparation and sequencing protocol with sequencing instructions can be found in this link.

#### Bacterial whole genome sequencing

For each LoopSeq Bacterial Genome kit, up to 8 samples were processed in multiplex and ~40,000~5 kb molecules were sequenced per sample (~320 k molecules per library). 320M PE150 reads (160M clusters passing filter) were used for each sequencing run, yielding ~50 gigabases (Gb) of data. The complete sample preparation and sequencing protocol with sequencing instructions can be found in this link.

### Short read coverage of LoopSeq synthetic long reads (SLRs)

In general, greater short-read coverage of each SLR will result in a higher fraction of complete SLRs (i.e., SLRs that span the full targeted amplicon) and a lower error rate. Here, we evaluated LoopSeq data with an average of 300 150bp reads per full-length 16S read (30× coverage), which is the recommended short-read coverage in the manufacturer protocols. The minimal number of 150bp short reads required to assemble a full-length 1.5 kb 16S rRNA gene using the LoopSeq SPADES workflow is 30 (3× coverage), but this would result in a significantly higher per-base error rate. Correspondingly, higher than 30× short-read coverage per SLR would be expected to produce per-base error rates even lower than those reported here.

### Assembly of SLRs

Loop Genomics maintains a cloud-based platform for processing raw short-reads prepared with a LoopSeq kit into assembled SLR contigs. Within this pipeline, short-reads are trimmed using Trimmomatic [7] to remove adapter sequences before they are de-multiplexed based on their Loop Sample Index, which groups them by the sample from which they originated. Within a grouped sample, short-reads are next binned by UMI such that those with the same UMI are processed collectively through SPADES [4]. Reads sharing the same UMI are derived from the same original molecule, with each read covering a different region of the sequence. With enough short-read data to cover the full length of a long DNA molecule, it is possible to assemble the original long DNA molecule by linking overlapping short-reads through their shared sequence, and then arranging the reads in the correct order to rebuild the original 16S/18S-ITS/Genomic molecule sequence. Assembly attempts with fewer reads result in shorter SLRs with lower accuracy.

### Amplicon bioinformatics

The raw LoopSeq synthetic long reads (SLRs) were subjected to further quality filtering, denoising, and chimera removal using the dada2 R package, largely following the long-read workflow previously established for PacBio long-read amplicon sequencing [12]. Briefly, SLRs were screened for the presence of both forward and reverse primers of the full length 16S gene, and truncated to the region between those primers. Primer-free sequences were filtered based on the total expected errors (Flvyberg and [18]). The relationship between the quality scores and the error rates was learned from the data, and the denoised amplicon sequence variants (ASVs) were inferred using the DADA2 algorithm [10].

Standard data processing used the default parameters for long-reads described in [12]. High-sensitivity parameters appropriate for LoopSeq data were also developed and used in several analyses. The key differences between the high-sensitivity and default parameters were that the probability threshold for detecting new ASVs was made less stringent (OMEGA_A=1e−10), and the option to directly detect singleton sequences was enabled (DETECT_SINGLETONS=TRUE).

### Characterizing synthetic long-reads by error type

After screening for and removing primers, a sliding window comparison was made between every synthetic long-read (SLR) present in the Zymo mock community data and the 27 “reference” sequences corresponding to the unique full-length 16S rRNA gene alleles present in each mock community strain. For each window of 50 nts (step size of 10 nts), the most similar reference sequence(s) were recorded, and a strain-level assignment for that SLR-window was made if all of the most similar reference sequences belonged to the same strain. Otherwise, no strain-level assignment was made for that window.

Two types of structural errors — chimeras and introgressions — were assigned based on the results of the sliding window comparison. A chimera assignment was made if the best match to the left-hand side and the right-hand side of the SLR were from different strains. An introgression assignment was made if an internal segment was assigned to a different strain than the rest of the SLR. Windows in which no strain assignment was made were ignored. More complex patterns (e.g., SLRs that contained windows assigned to three or more different strains) were designated as complex errors. Finally, SLRs that had a consistent strain-level assignment throughout, but that had up to three mismatches with the closest reference sequence, were assigned as point errors.

### Comparing LoopSeq and PacBio 16S sequences

The currently recommended 16S primer sets used in the LoopSeq and PacBio sequencing reported here are nearly identical. The PacBio forward primer was “AGRGTTYGATYMTGGCTCAG” and the PacBio reverse primer was “RGYTACCTTGTTACGACTT.” The LoopSeq forward primer was “AGAGTTTGATCMTGGC” and the reverse primer was “TACCTTGTTACGACTT.” These primer sets amplify nearly identical amplicons, up to a 4 nucleotide difference between the extent of the PacBio and LoopSeq forward primer sequences. As a result, we were able to directly merge the sequences generated by these two different technologies after trimming 4 base pairs off the start of the LoopSeq reads.

### Assessing conserved/variable status of substitutions

The DADA2 algorithm provides a complete description of the nucleotides differences that distinguish each ASV from the “sibling” ASV from which it was divided. The ssu-align method (http://eddylab.org/software/ssu-align/) was used to align each “sibling” ASV to a model for the bacterial 16S rRNA gene, and thereby to classify each nucleotide in the “sibling” ASV as either conserved or variable. Together, these two sources of information allowed the distinguishing substitutions for each ASV identified by DADA2 to be classified as occurring in conserved or variable positions. The null expectation of the ratio of conserved to variable positions was determined by randomizing the positions of distinguishing substitutions.

### Species assignment for potential foodborne pathogens

Six meat samples were non-randomly chosen to ensure multiple foodborne pathogen species were present from a broader library of foodborne pathogen surveillance samples collected as part of the NARMS Retail Meat project. Species-level assignments were made by taking a consensus of BLAST results for each ASV. That is, sequences were BLAST-ed against nt, excluding uncultured/environmental accessions. The species designations of all BLAST hits sharing the top score were collated. If all top-hit species designations agreed, a species assignment was made. Accessions with no species designation were ignored.

### Code availability and reproducible analysis

Rmarkdown code to reproduce the results described in this paper is available at https://github.com/benjjneb/LoopManuscript

## Supplementary Information

**Additional file 1: Figure S1.** The abundances of all ASVs identified by LoopSeq and default DADA2 in the Zymo mock community. All abundances are scaled to the abundance of the corresponding genome in the amplified 16S rRNA gene data. The near-integer values of all genome-scaled abundances are consistent with each ASV representing a unique allele present in the multiple copies of the 16S rRNA gene present in the genomes of these strains.

## Data Availability

All sequencing data are available from the SRA under BioProject Accession PRJNA644197. Rmarkdown code to reproduce the results described in this paper is available at https://github.com/benjjneb/LoopManuscript. The exact code that produced the accepted paper is available as the “Publication” release under doi: TBD.
