# Supplementary material for: Ultra-accurate microbial amplicon sequencing with synthetic long reads
Source: Microbiome. 2021 Jun 5;9:130. doi: 10.1186/s40168-021-01072-3 (PMC8179091; doi:10.1186/s40168-021-01072-3)
Supplement: Supplementary file 2 — Additional file 1: Figure S1. The abundances of all ASVs identified by LoopSeq and default DADA2 in the Zymo mock community. All abundances are scaled to the abundance of the corresponding genome in the amplified 16S rRNA gene data. The near-integer values of all genome-scaled abundances are consistent with each ASV representing a unique allele present in the multiple copies of the 16S rRNA gene present in the genomes of these strains. [file 40168_2021_1072_MOESM2_ESM.docx]

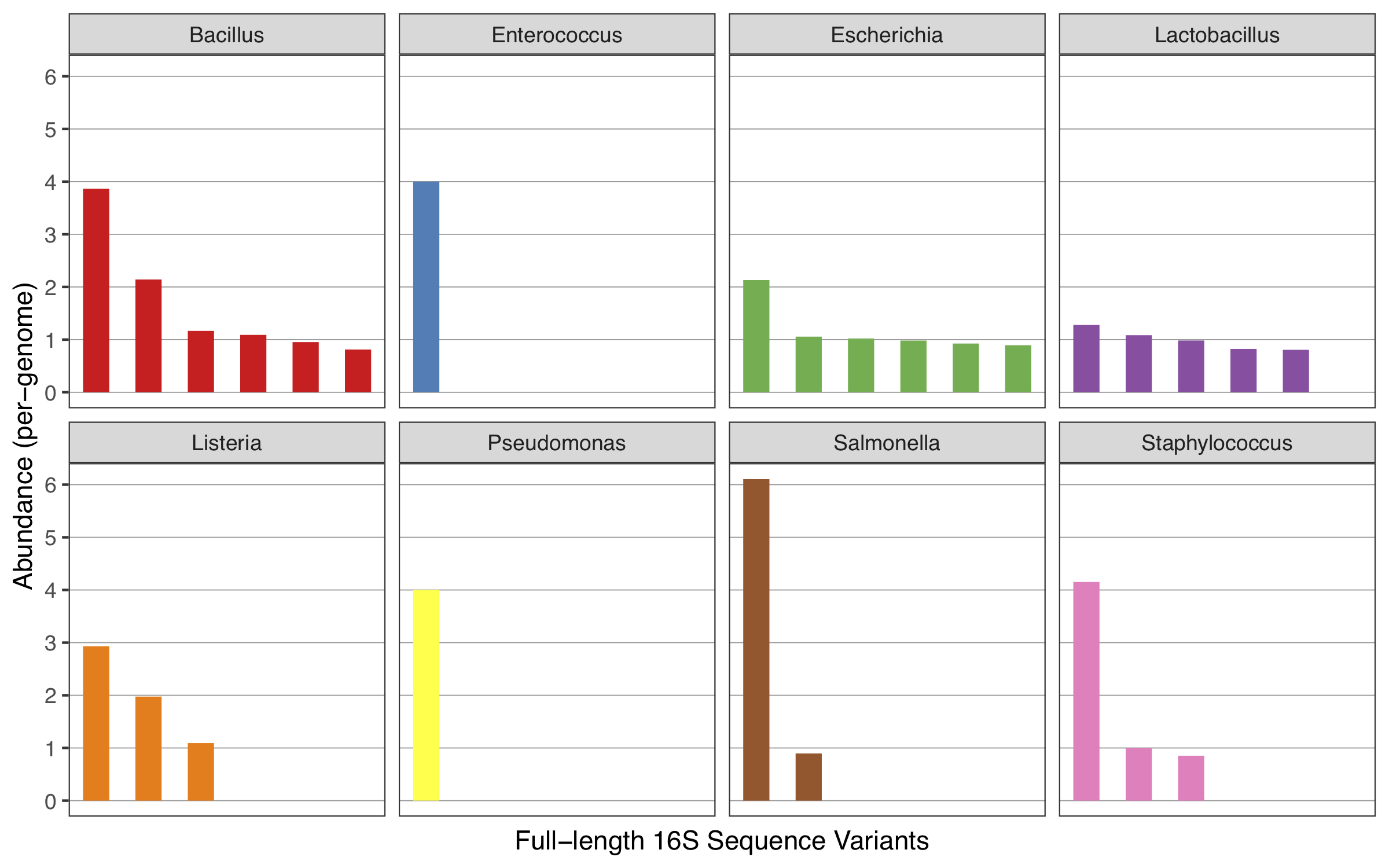


*Figure S1: The abundances of all ASVs identified by LoopSeq and default DADA2 in the Zymo mock community. All abundances are scaled to the abundance of the corresponding genome in the amplified 16S rRNA gene data. The near-integer values of all genome-scaled abundances are consistent with each ASV representing a unique allele present in the multiple copies of the 16S rRNA gene present in the genomes of these strains.*
